# Concurrent Presentation of Bipolar Affective Disorder and Recent Alcohol Cessation: Diagnostic Challenges in Resource Limited Setting

**DOI:** 10.24248/eahrj.v9i2.841

**Published:** 2025-12-24

**Authors:** Milton Anguyo, Emmanuel Alyoomu, Habert Aziku, Joel Masette

**Affiliations:** a Faculty of Medicine, Gulu Universityx, Gulu, Uganda

## Abstract

**Background:**

Bipolar Affective Disorder (BPAD) frequently coexists with Alcohol Use Disorder (AUD), creating diagnostic complexities, particularly when manic symptoms occur close to alcohol cessation. Accurate differentiation between primary mania and substance induced symptoms is critical for appropriate management.

**Case Presentation:**

We describe a male in his early 30s with a two week history of elevated mood, irritability, reduced need for sleep, increased goal directed activity, and psychotic features, occurring shortly after abrupt cessation of chronic alcohol use. Although the patient initially reported stopping alcohol 3 months prior, collateral history revealed continued use until days before presentation. A previous untreated manic episode three years earlier strongly suggested primary BPAD. Diagnostic evaluation using DSM-5-TR criteria and the Mini International Neuropsychiatric Interview (MINI) supported a diagnosis of BPAD, current manic episode with psychotic features.

**Management and Outcome:**

Treatment comprised carbamazepine, chlorpromazine, and a short diazepam course for anxiolysis. Psychoeducation and motivational interviewing addressed both the mood episode and AUD. The patient achieved full symptomatic remission and remained abstinent on follow-up.

**Conclusion:**

This case emphasizes the importance of longitudinal psychiatric history, collateral information, and standardized diagnostic tools in distinguishing primary BPAD from alcohol related presentations in low resource settings.

## BACKGROUND

Bipolar Affective Disorder (BPAD) is a chronic psychiatric illness characterized by episodic mania, hypomania, and depression. Globally, BPAD has an estimated prevalence of 1% to 3% and represents a leading cause of disability.^1^

Comorbidity with Alcohol Use Disorder (AUD) is common, with up to 70% of individuals with BPAD reporting lifetime problematic alcohol use.^2^ AUD complicates diagnostic clarity, worsens prognosis, increases relapse rates, and predicts poor adherence to treatment.

Alcohol Withdrawal Syndrome (AWS) typically develops within hours of cessation of heavy and prolonged use, presenting with tremors, autonomic hyperactivity, insomnia, anxiety, and, in severe cases, delirium tremens.^3^ Symptom severity and timing in relation to cessation are essential in evaluating whether alcohol withdrawal contributes to psychiatric symptoms.

Differentiating between a primary manic episode and alcohol-induced mood symptoms requires detailed longitudinal history, collateral information, objective diagnostic tools, and evaluation of the temporal relationship between alcohol cessation and symptom onset. The DSM-5-TR and MINI are validated tools guiding diagnostic accuracy in such complex presentations.^4–6^

Here, we report a case of a young adult male presenting with manic and psychotic symptoms shortly after abrupt alcohol cessation, in a context complicated by chronic AUD and an unclear timeline of abstinence.

## CASE PRESENTATION

A male in his early 30s was brought to the mental health unit with a 2 week history of behavioral and mood changes. These included excessive happiness, irritability, rapid and pressured speech, decreased need for sleep, increased goal-directed activity, financial recklessness, heightened anxiety, and both auditory and visual hallucinations (including seeing deceased individuals). There were no features suggestive of a depressive episode preceding the current illness.

### Past Psychiatric History

Three years earlier, he experienced a similar untreated episode, lasting several months, characterized by reduced sleep, irritability, increased talkativeness, heightened energy, and risky behaviors. Symptoms resolved spontaneously. He never received psychiatric evaluation or medication.

### Substance Use History

The patient reported 11 years of heavy daily alcohol consumption, including beer and local spirits. He initially stated that he had stopped drinking alcohol 3 months before presentation. However, his attendant clarified that although he reduced his intake at that time, he continued consuming alcohol until 3 to 5 days before admission, when he abruptly stopped.

This corrected timeline was essential in differentiating AWS from primary mood pathology.

### Family History

There was a positive history of mental illness in two of his children. No family history of substance use disorders was reported.

### Physical Examination

General physical examination revealed no autonomic instability, tremors, diaphoresis, or hepatic stigmata. Vital signs were stable.

### Mental State Examination

Patient appeared well groomed, cooperative and subjectively “happy” but irritable, expansive and labile. He had auditory and visual hallucinations, and grandiose delusions with his speech being tangential and pressured. Although his orientation was intact, the insight was absent and his attention and concentration were impaired.

### Laboratory Investigations

The patient had normal results for Complete Blood Count (CBC), Liver Function Tests (LFTs), Renal Function Tests (RFTs) and Random Blood Sugar, and tested negative for HIV. In short no metabolic abnormalities or infectious processes were identified.

### DSM-5-TR Evaluation

The patient met criteria for a Manic Episode, which included elevated and irritable mood, reduced need for sleep, pressure of speech, grandiosity, increased goal directed activity, psychotic features for a duration of >1 week and significant functional impairment. Thus, the diagnosis of Bipolar I Disorder, Current Episode Manic with Psychotic Features was confirmed.

The MINI v7.0 similarly confirmed presence of Manic Episode with psychotic features, Alcohol Use Disorder, but no criteria met for schizophrenia, schizoaffective disorder, or primary psychotic disorder. Although the corrected timeline showed alcohol cessation a few days before onset, the patient lacked hallmark AWS features which include tremors, autonomic hyperactivity, seizures, disorientation or fluctuating consciousness. Psychotic symptoms were mood congruent, supporting primary mania and not withdrawal-associated delirium. Thus final diagnoses consisted of Bipolar Affective Disorder, Current Manic Episode with Psychotic Features and Alcohol Use Disorder. The differential diagnoses included Alcohol-Induced Psychotic Disorder and AWS.

### Treatment

The patient was given Carbamazepine 200 mg twice daily for mood stabilization, Chlorpromazine 400 mg twice daily for control of psychosis and agitation, Diazepam short course (5–10 mg daily tapered over 6 days) for anxiolysis, not AWS detoxification. He was also subjected to psychoeducation on BPAD and relapse prevention. A motivational interviewing for AUD was conducted and sleep hygiene counselling provided. Family members were involved for monitoring and support.

### Outcome and Follow-Up

The patient improved significantly after initiation of treatment. Manic and psychotic symptoms resolved within two weeks. He was discharged stable, continued outpatient follow-up, and remained abstinent from alcohol for six months with good functional recovery.

## DISCUSSION

The present case illustrates the complex diagnostic challenges encountered when manic symptoms emerge in close proximity to alcohol cessation, particularly in low-resource settings where structured diagnostic tools and specialist services may be limited. Rather than merely restating the findings, this discussion integrates the patient's presentation with existing literature and accepted principles in psychiatric diagnostic practice.

First, the overlap between symptoms of Bipolar Affective Disorder (BPAD) and Alcohol Withdrawal Syndrome (AWS) is well documented, with both conditions potentially presenting with irritability, psychomotor agitation, insomnia, anxiety, and perceptual disturbances.^3^ However, international evidence emphasizes that AWS typically develops within 6–24 hours after cessation of heavy alcohol use and often includes autonomic hyperactivity, tremors, diaphoresis, and fluctuating consciousness, features absent in our patient.^3^⁻^8^ Recent studies show that withdrawal-related psychosis tends to be transient, fluctuating, and accompanied by disorientation, while psychotic features in primary mania are usually mood-congruent and sustained, as was the case here.7 This distinction aligns our findings with the broader literature supporting mood-congruent psychosis as a strong indicator of primary BPAD rather than substance-induced syndromes.

Second, the case reinforces the importance of longitudinal psychiatric history in diagnostic decision-making. According to DSM-5-TR diagnostic principles, a prior spontaneous manic episode especially one occurring independently of substance use strongly argues for primary BPAD rather than a substance-induced mood disorder.^4^ The untreated manic episode three years prior provided a critical historical anchor, consistent with findings from Goodwin et al. and Berk et al., who highlight the predictive value of prior episodes in differential diagnosis and relapse prevention.^1 7^ Thus, the patient's history fits well within established diagnostic frameworks.

Third, collateral history remains a cornerstone of good psychiatric practice, especially when substance use complicates clinical presentation. In resource-limited settings, patients may underreport alcohol use due to stigma, impaired insight, or memory distortion. This patient's inaccurate initial report of 3-month abstinence could easily have misled clinicians. Literature from Schuckit and others emphasizes the unreliability of self-report among individuals with active or recent alcohol misuse, supporting the need for corroborative information.8 The attendant's clarification therefore not only enhanced diagnostic precision but also reflects established best practice in addiction psychiatry.

Fourth, the use of structured diagnostic instruments such as the DSM-5-TR criteria and the Mini International Neuropsychiatric Interview (MINI) strengthened diagnostic reliability. Tools like the MINI have been validated for use in both high- and low-resource contexts and are particularly useful where specialist psychiatric assessment may be unavailable.^5^ Their application in this case exemplifies the “community of practice” approach recommended in low-resource mental health care, utilizing structured, standardized instruments to support accurate diagnosis when clinical complexity is high.

Finally, integrated treatment of BPAD comorbid with Alcohol Use Disorder (AUD) reflects contemporary best practice. The combination of a mood stabilizer, an antipsychotic, brief benzodiazepine support, motivational interviewing, and psychoeducation aligns with multidisciplinary treatment models advocated in the literature.^1 7 8^ Evidence suggests that addressing both conditions concurrently improves adherence, reduces relapse rates, and enhances long-term functional outcomes.^2^ The patient's sustained remission and six-month abstinence following integrated treatment further support these findings.

Overall, the diagnostic process and treatment approach in this case reflect established clinical practice and previous research, emphasizing that accurate diagnosis of mania in the context of recent alcohol cessation requires careful temporal analysis, collateral information, historical psychiatric context, structured diagnostic tools, and integrated management. This case therefore contributes to existing literature by demonstrating how these principles can be effectively implemented even in low-resource environments.

## CONCLUSIONS, AND RECOMMENDATIONS

This case underscores the need for clinicians to maintain a high index of suspicion for primary Bipolar Affective Disorder when manic symptoms occur independently or recur outside the context of substance use. A detailed longitudinal psychiatric history, supported by collateral information, plays an essential role in ensuring diagnostic accuracy, particularly in settings where patients may underreport alcohol use or where insight is impaired. The use of structured diagnostic tools such as the DSM-5-TR criteria and the MINI further strengthens diagnostic reliability and should be routinely incorporated into clinical assessments, especially in low-resource environments.

The presentation also highlights that although alcohol cessation can produce symptoms such as anxiety, insomnia, and irritability, it rarely results in sustained psychotic mania. Careful attention to the temporal sequence of events and the presence or absence of classic alcohol withdrawal features is therefore crucial in distinguishing AWS or substance-induced disorders from primary mood pathology.

From this case, an important conclusion emerges: effective management of comorbid BPAD and Alcohol Use Disorder requires an integrated, multidisciplinary approach that addresses both conditions concurrently. Pharmacological treatment should be paired with psychosocial interventions, motivational interviewing, and family involvement to enhance recovery and reduce relapse risk. Strengthening clinical training in dual-diagnosis assessment and expanding access to validated diagnostic instruments would further improve outcomes in similar contexts.

Overall, this case demonstrates that accurate diagnosis depends on synthesizing clinical history, collateral information, structured assessments, and careful interpretation of symptom patterns. Scaling up these practices across mental health services in resource-limited settings is strongly recommended to enhance diagnostic precision and treatment effectiveness.
